# Supplementary material for: Anticancer Effect of Hemin through ANO1 Inhibition in Human Prostate Cancer Cells
Source: Int J Mol Sci. 2024 May 30;25(11):6032. doi: 10.3390/ijms25116032 (PMC11172662; doi:10.3390/ijms25116032)
Supplement: Supplementary file 1 [file ijms-25-06032-s001.zip › ijms-2989346-supplementary.pdf]

Supplementary Materials Data

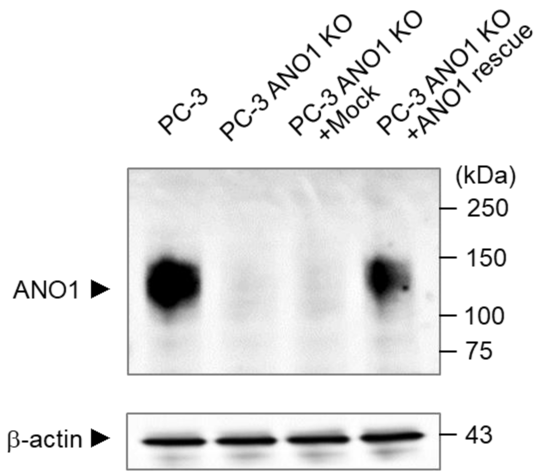

Figure S1. Expression levels of ANO1 protein. ANO1 protein expression levels were evaluated in PC-3 cells, PC-3 ANO1 KO, and PC-3 ANO1 KO cells subjected to either mock transfection or ANO1 transfection.

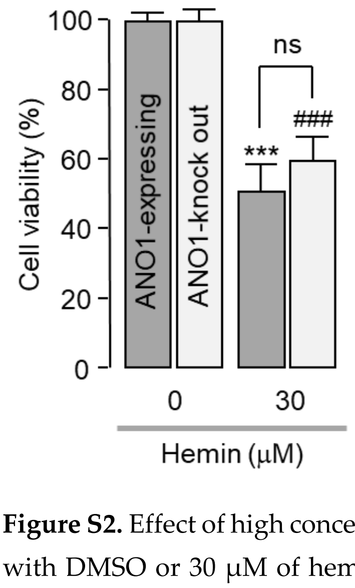

Figure S2. Effect of high concentration of hemin on cell viability in PC-3, and PC-3 ANO1 KO cells. Cells were incubated with DMSO or 30 μM of hemin for 72 h, and medium was replaced every 24 h (mean ± S.D., n = 5). *** $p < 0.001$ vs. ANO1-expressing control; ### $p < 0.001$ vs. ANO1 KO control; ns: not significant.
